# Interpretation of Antibiotic Trials in Pediatric Pneumonia

**DOI:** 10.1001/jamanetworkopen.2023.54470

**Published:** 2024-02-02

**Authors:** Susan C. Lipsett, Alexander W. Hirsch, Richard G. Bachur, Mark I. Neuman

**Affiliations:** 1 Division of Emergency Medicine, Boston Children’s Hospital, Boston, Massachusetts; 2 Department of Pediatrics, Harvard Medical School, Boston, Massachusetts

## Abstract

This cohort study assesses radiographic evidence of pneumonia and antibiotic use in children with clinically suspected community-acquired pneumonia.

## Introduction

Recent randomized clinical trials have compared antibiotic regimens of varying intensity for the treatment of pediatric community-acquired pneumonia (CAP).^1,2,3,4^ These trials identified no advantage to high-dose vs low-dose amoxicillin^3^ or a longer vs shorter duration of antibiotic therapy^1,2,3,4^ with respect to rates or timing of clinical improvement. However, most of these trials enrolled children based solely on clinical criteria or clinician judgment.^2,3,4^ Given the challenges of diagnosing pediatric pneumonia clinically, it is likely that most children included in studies with clinically suspected CAP did not have radiographic pneumonia and thus would have improved regardless of antibiotic therapy.

## Methods

We performed an analysis of an ongoing prospective cohort study of children presenting to a tertiary-care pediatric emergency department (ED) and undergoing chest radiography (CXR) for clinically suspected CAP.^5^ The Boston Children's Hospital Institutional Review Board approved this cohort study. Participants provided verbal informed consent. We followed the STROBE reporting guideline.

To identify participants most similar to those in recent antibiotic dose and duration trials, the cohort was restricted to generally healthy^6^ children aged 3 months to 6 years who were not pretreated with antibiotics. Children were classified according to whether they satisfied the inclusion criteria for the Community-Acquired Pneumonia: A Randomized Controlled Trial (CAP-IT).^3^ Specifically, inclusion required all of the following: cough within the prior 96 hours; fever within the prior 48 hours; and signs of labored breathing, focal chest signs, or lobar pneumonia on CXR. Children with bilateral wheezing and no focal chest signs were excluded.^3^ All children in the present cohort underwent CXR for evaluation for pneumonia, whereas the CAP-IT trial^3^ and other studies^2,4^ allowed inclusion based on clinical judgment alone.

Based on CXR interpretations by board-certified pediatric radiologists as part of routine clinical care, radiographs were classified as positive, equivocal, or negative for the presence of pneumonia. Clinical outcomes were assessed using structured follow-up 7 to 14 days after the ED visit. We calculated the percentage of children who recovered from their illness without antibiotic use. Data analysis was performed from May to November 2023 using Stata SE 16 (StataCorp LLC).

## Results

Between May 2015 and November 2022, 1252 patients aged 3 months to 6 years (median [IQR] age, 2.1 [1.2-3.7] years; 566 females [45.2%], 686 males [54.8%]) were enrolled. Five hundred seven patients (40.5%) met the CAP-IT trial inclusion criteria (Table 1). Of these, 154 (30.3%) had radiographic pneumonia, 49 (9.7%) had equivocal radiographs, and 304 (60.0%) had no radiographic pneumonia. Among children without radiographic pneumonia, 252 (82.9%) were discharged without antibiotics, 5 (2.0%) of whom were diagnosed with pneumonia within 2 weeks after discharge (Table 2). A total of 245 children (48.3%) who satisfied the inclusion criteria recovered without antibiotics.

**Table 1.** Patient Characteristics

Characteristic	No. (%) [n = 1252]
Age, median (IQR), y	2.1 (1.2-3.7)
<1	248 (19.8)
1-2	331 (26.4)
2-5	564 (45.1)
5-6	109 (8.7)
Sex	
Female	566 (45.2)
Male	686 (54.8)
Hospitalized	282 (22.7)
CXR findings	
Definite pneumonia	150 (12.0)
Probable pneumonia	35 (2.8)
Equivocal	64 (5.1)
Unlikely pneumonia	52 (4.2)
No pneumonia	951 (76.0)
Inclusion criteria (adapted from CAP-IT)	
Cough within previous 96 h	1158 (92.5)
Fever within previous 48 h	1033 (82.5)
Signs of labored breathing	
Retractions	391 (31.2)
Respiratory distress^a^	297 (23.7)
Focal lung findings^b^	
Crackles with asymmetry	224 (17.9)
Focal decreased breath sounds	170 (13.6)
CXR demonstrating pneumonia^c^	154 (12.3)
Met criteria for pneumonia^d^	507 (40.5)

Abbreviations: CAP-IT, Community-Acquired Pneumonia: A Randomized Controlled Trial; CXR, chest radiography.

^a^ Includes nasal flaring and abdominal breathing.

^b^ Focal dullness to percussion not assessed in this cohort.

^c^ As classified by board-certified pediatric radiologist.

^d^ Excluding 74 patients (5.9%) with bilateral wheezing without focal chest signs.

**Table 2.** Characteristics of Patients With Equivocal or Negative Chest Radiographs Diagnosed With Pneumonia Within 2 Weeks

Age	Sex	Duration of fever, d	Temperature in ED, °C	Oxygen saturation in ED, %	Disposition from ED	Hospitalized at follow-up?	Clinical synopsis
7 mo	Female	1-3	39.0	100	Discharged	No	Returned to ED 3 d later with persistent symptoms. Chest radiograph showed bronchial wall thickening and hyperinflated lungs suggestive of viral infection but with additional patchy opacities raising concern for superimposed pneumonia. Treated with amoxicillin.
8 mo	Male	<1	38.2	100	Discharged	No	Diagnosed with pneumonia by primary care physician within 3 d of ED visit. Treated with amoxicillin.
21 mo	Female	<1	40.3	100	Discharged	No	Returned to ED 3 d later with persistent symptoms. Chest radiograph showed peribronchial cuffing and hyperinflated lungs suggestive of viral infection but with additional rounded opacity in the right middle lobe compatible with superimposed pneumonia. Treated with amoxicillin.
2 y	Female	1-3	39.4	95	Discharged	No	Diagnosed with pneumonia by primary care physician within 3 d of ED visit. Treated with amoxicillin.
2 y	Female	1-3	37.6	98	Discharged	No	Diagnosed with pneumonia by primary care physician 4-6 d following ED visit. Treated with amoxicillin.

Abbreviation: ED, emergency department.

## Discussion

In this analysis of prospectively collected data mirroring the inclusion criteria for a recent antibiotic trial of pediatric CAP, we observed that only 30.3% of children who met the criteria had positive radiographs, which is similar to the rate of lobar radiographic pneumonia in the CAP-IT trial (34%).^3^ Additionally, 48.3% of patients recovered from their illness without antibiotic use. Prior data suggest that most children with suspected pneumonia and negative radiographs recover without antibiotics.^5^

A study limitation is the lack of a true gold standard for pneumonia diagnosis in children. We hypothesize that most antibiotic trials in pediatric CAP include many patients without pneumonia and thus may be underpowered to identify true differences in outcomes for children with bacterial pneumonia. These important pragmatic trials support reducing the intensity of antibiotic use in children with respiratory illness. However, widespread use of deintensified antibiotic regimens for all children with pneumonia, particularly those with lobar consolidations, may be premature given the paucity of evidence supporting their efficacy for these populations.

## Notes

Supplement.
Data Sharing Statement
